# Renal impairment in a rural African antiretroviral programme

**DOI:** 10.1186/1471-2334-9-143

**Published:** 2009-08-28

**Authors:** Cara Franey, Deborah Knott, Till Barnighausen, Martin Dedicoat, Ahmed Adam, Richard J Lessells, Marie-Louise Newell, Graham S Cooke

**Affiliations:** 1 Africa Centre for Health and Population Studies, University of KwaZulu Natal, South Africa; 2 Hlabisa Hospital, KwaZulu Natal, South Africa; 3 School of Public Health, Harvard, USA; 4 Ngwelezane Hospital, Empangeni, South Africa; 5 Institute of Child Health, UCL, UK; 6 Division of Medicine, Imperial College, UK

## Abstract

**Background:**

There is little knowledge regarding the prevalence and nature of renal impairment in African populations initiating antiretroviral treatment, nor evidence to inform the most cost effective methods of screening for renal impairment. With the increasing availability of the potentially nephrotixic drug, tenofovir, such information is important for the planning of antiretroviral programmes

**Methods:**

(i) Retrospective review of the prevalence and risk factors for impaired renal function in 2189 individuals initiating antiretroviral treatment in a rural African setting between 2004 and 2007 (ii) A prospective study of 149 consecutive patients initiating antiretrovirals to assess the utility of urine analysis for the detection of impaired renal function. Severe renal and moderately impaired renal function were defined as an estimated GFR of ≤ 30 mls/min/1.73 m^2 ^and 30–60 mls/min/1.73 m^2 ^respectively. Logistic regression was used to determine odds ratio (OR) of significantly impaired renal function (combining severe and moderate impairment). Co-variates for analysis were age, sex and CD4 count at initiation.

**Results:**

(i) There was a low prevalence of severe renal impairment (29/2189, 1.3% 95% C.I. 0.8–1.8) whereas moderate renal impairment was more frequent (287/2189, 13.1% 95% C.I. 11.6–14.5) with many patients having advanced immunosuppression at treatment initiation (median CD4 120 cells/μl). In multivariable logistic regression age over 40 (aOR 4.65, 95% C.I. 3.54–6.1), male gender (aOR 1.89, 95% C.I. 1.39–2.56) and CD4<100 cells/ul (aOR 1.4, 95% C.I. 1.07–1.82) were associated with risk of significant renal impairment (ii) In 149 consecutive patients, urine analysis had poor sensitivity and specificity for detecting impaired renal function.

**Conclusion:**

In this rural African setting, significant renal impairment is uncommon in patients initiating antiretrovirals. Urine analysis alone may be inadequate for identification of those with impaired renal function where resources for biochemistry are limited.

## Background

Renal disease is an important complication of both HIV infection [1] and antiretroviral treatment [2]. In a Western setting HIV-related renal disease appears to be more common in populations of African descent [3,4] and as antiretroviral roll-out continues across sub-Saharan Africa, the prevalence and nature of renal impairment in HIV positive individuals from African populations is of increasing importance [5]. However, little data exists on the prevalence of renal impairment before or during antiretroviral treatment in rural African areas, and in particular there is very little data from non-specialist centres or research programmes.

Such information is important for individual patient care, the programmatic choice of antiretrovirals and strategies for monitoring toxicity. In most roll-out programmes in Sub-Saharan Africa, stavudine and lamivudine are first line medications and should have their dosages reduced in the setting of renal impairment; without doing so the chance of toxicity may be increased [6]. Similarly, patients with pre-existing renal disease are likely to require closer monitoring once treatment is initiated.

With an ongoing discussion about the possibility of introducing tenofovir as first line treatment in countries such as South Africa (which is home to approximately one in six of the world's HIV positive population[7]), following it's recommendation by WHO [8], the prevalence of renal impairment takes on additional importance as patients with pre-existing renal impairment might either require alternative treatments or be at greater risk of toxicity and require more intensive monitoring [9].

In many resource-poor settings within Sub-Saharan Africa, the diagnosis of renal impairment using blood biochemistry is not a routine matter. Point of care tests, particularly urine analysis, offer a possible method of screening patients at risk of renal disease with the potential for targeting high risk groups for more intensive monitoring.

We set out to establish the prevalence of, and risk factors for, renal impairment in a large rural South African antiretroviral programme and, in addition, to study whether the presence of renal impairment could be predicted by urine analysis.

## Methods

Patients were recruited from a public sector service in the rural Hlabisa sub-district of KwaZulu Natal, South Africa. The local antiretroviral programme began in late 2004 as a partnership between the Department of Health and the Africa Centre for Health and Population Studies, Somkhele. The service is run according to South African National guidelines and thus patients become eligible for treatment on the basis of a CD4 count below 200 cells/ml or WHO Stage IV disease. Prior to initiation with stavudine, lamivudine and either efavirenz or nevirapine, patients routinely have baseline blood biochemistry performed, including serum creatinine.

Details of age, sex, weight, CD4 count, and serum creatinine were collected where available from medical records from all 2500 patients initiating treatment between November 2004 and June 2007. Patients who had previously received combination antiretrovirals and patients under the age of 16 years were excluded.

GFR was estimated (eGFR) with the widely-used, abbreviated four variable MDRD method [2]. All patients were of Zulu ethnicity and thus for the MDRD calculation of the prevalence of renal impairment, the ethnicity correction factor of 1.21 was excluded on the assumption that the population would not differ from the population of black South Africans for whom this method was recently validated in a hospitalised population [10]. Cockcroft-Gault estimations were not performed as height measurements, required to calculate BSA, were not available for the majority of individuals studied.

Severe renal impairment was defined as an estimated GFR (eGFR) <30 mls/min/1.73 m^2^, moderate renal impairment as an eGFR of 30–60 mls/min/1.73 m^2 ^and mild renal impairment as an eGFR 60–90 mls/min/1.73 m^2^. Significant renal impairment was defined as the combination of these two (i.e. those with eGFR less than or equal to 60 mls/min/1.73 m^2^) as this population might require dose alteration to antiretroviral treatments.

Risk factors for renal impairment were estimated with logistic regression using the Stata10 package (StataCorp, Texas). Significant renal impairment (defined above) was the outcome variable. Demographic variables were included in the model, along with CD4 count at initiation (available for 1909 individuals).

In a separate analysis more detailed clinical data were recorded for 149 consecutive individuals initiating treatment. In addition to baseline demographics, single readings of blood pressure, random capillary glucose and urinalysis were made. One individuals was excluded from analysis as recorded to be menstruating. Urine analysis was performed using Multistix™ (Bayer, Germany) recording significant proteinuria (≥ 1+) and haematuria (≥ 1+). Individual informed consent was obtained from all participants in the programme to allow use of anonymised routine clinical data in research. Ethical approval was obtained from the ethics committee of University of KwaZulu Natal and the research office of KwaZulu Natal Department of Health.

## Results

### i) Prevalence and risk factors for renal impairment

192 individuals were either aged under 16 or had previously received combination antiretrovirals and were excluded. 119 had no record of baseline creatinine available and were excluded, leaving 2189 individuals for analysis.

1505 individuals were female (68.8%) and 684 (31.2%) male. Other baseline characteristics are shown in Table 1. Estimates of the proportion of patients with severe, moderate, mild reductions in eGFR (as defined above) are in Table 2. Within the most severe category, 8/29 had eGFR of less than 15 mls/min/1.73 m^2 ^and 21/29 15–30 mls/min/1.73 m^2^. If the ethnicity factor of 1.21 had been applied the numbers of patients with categorised with renal impairment were 18/2189 (0.8%) with eGFR<30 mls/min/1.73 m^2^, 110/2189 (5.0%) with eGFR 30–60 mls/min/1.73 m^2^.

**Table 1:** Baseline characteristics of initiating cohort studied

	Median	IQR (25–75)
Age (yrs)	36	30–43
Weight (kg)	59	52.1–67.5
Height (cm)	161	156–167
CD4 (cells/ul)	119	59–175

**Table 2:** GFR estimated by abbreviated MDRD methods (without ethnicity correction)

		N	%	95% CI
0–29		29	1.3	0.8–1.8
30–59		287	13.1	11.6–14.5
60–89		1250	57.1	55.0–59.2
90 or more		623	28.4	26.6–30.3

		2189		

For logistic regression, significant renal impairment (defined above) was used as the outcome variable. Dependent variables were age, gender and CD4 count. Univariably, male gender was significantly associated with outcome (OR 1.57, 95% CI 1.19–2.08 *P *< 0.01). Age and CD4 count were also associated with outcome both as continuous variables; age (OR 1.08 95% CI 1.07–1.09, *P *< 0.01), CD4 count (OR 0.99 CI 0.996–0.999, *P *= 0.041) and when coded as categorical values with age over 40 (OR 4.21, 95% CI 3.27–5.42 *P *< 0.01) or CD4 count <100 (OR 1.16 95% CI 0.90–1.49 *P *= 0.21). Categorical variables only were taken forward for the multivariable analysis the results, results are shown in Table 3.

**Table 3:** Multivariable logistic regression

	aOR1	P	95% C.I.
Age over 40	4.65	<0.01	3.54 – 6.10
Male	1.89	<0.01	1.39 – 2.56
CD4 <100	1.40	0.02	1.07 – 1.82

^1^Adjusted for other variables in the table

### ii) Sensitivity and specificity of urine analysis

In the consecutive series of 149 patients initiating antiretroviral therapy, the prevalence of renal impairment was similar to the larger cohort with 3/149 (2%) classified as severe renal impairment and 19/149 (12.9%) having moderate renal impairment. One patient had a blood pressure single reading with diastolic >90 mmHg and one patient had a random glucose of >11.1 but neither had significant renal impairment. 25/149 (16.7%) of individuals had evidence of haematuria, 39/149 (26.1%) had evidence of proteinuria, 53/149 (35.6%) had evidence of either proteinuria or haematuria and 63/149 (42.2%) had evidence of renal dysfunction (defined as either reduced eGFR and/or proteinuria/haematuria). Of those with proteinuria, 16/39 were recorded with 1+ protein, 18/39 with 2+ protein and 5/39 with 3+ protein.

The sensitivity of significant blood and protein on urine dipstix was assessed in a field setting. 6/22 (27.2%) patients with significant renal impairment had detectable protein and 7/22 (31.8%) detectable blood in their urine. 12/22 (54.5%) had either significant blood or protein. 33/127 (25.9%) of those without significant renal impairment had significant proteinuria whilst 18/127 (14.1%) had significant haematuria. Either significant proteinuria or haematuria was seen in 12/22 (54.5%) individuals with significant renal impairment and 41/127 (32.2%) without. Sensitivity, Specificty and positive predictive values for either the presence of blood, presence of protein or presence of either are shown in table 4.

**Table 4:** Sensitivity and specificity of urine analysis for detecting significantly impaired renal function

	Presence blood	Presence Protein	Presence either blood or protein
Sensitivity	0.31 (0.15–0.55)	0.27 (0.11–0.50)	0.55 (0.32–0.75)

Specificity	0.85 (0.78–0.91)	0.74 (0.65–0.81)	0.68 (0.59–0.76)

PPV	0.28 (0.13–0.49)	0.15 (0.06–0.31)	0.22 (0.13–0.19)

## Discussion

Over the coming years most people initiating antiretroviral treatment will do so in resource poor environments where laboratory investigations are limited and, in particular, in sub-Saharan Africa. Real world data from such settings is therefore important in prioritising the use of scarce resources for planning diagnostic, therapeutic and monitoring options. The data presented here represent the first published study to date of renal impairment from a rural African antiretroviral programme. There are reasons to think that impaired renal function might be common in such settings as African ethnicity and advanced immunosuppression [3,11] are identified risk factors for renal disease in a developed world setting. In this study we find severe renal impairment to be uncommon, even in a population starting treatment late in disease progression. Moderate renal impairment was more common with a prevalence of 14.4%.

The prevalence of significant renal impairment described here is slightly higher than seen in a recently reported Kenyan cohort of untreated patients who had a significantly higher CD4 counts [12]. However, the prevalence is lower than in a Ugandan study of home based care where more severe cases of renal disease had already been excluded [13] and where a similar association with increasing age was also observed. Direct comparisons have to be tempered by an understanding that there are significant differences in the populations studied. Additionally, whilst the best validated method for estimating eGFR in this population was used without a correction for ethnicity, this method has not been validated in other African populations for which it might be appropriate.

Of note, mildly impaired renal function was common. The terminology used here is not meant to imply that this is not significant. However, although common, such patients are unlikely to need changes to doses of medication or additional monitoring. Furthermore, moderate or mild renal impairment might well improve on antiretroviral therapy [14] and its relevance to programmatic decisions on drug choice and monitoring is therefore not clear.

These findings are likely to be representative of many patients starting treatment outside of specialist centres in rural Africa. A small number of individuals were excluded due to missing values of creatinine but there is no reason to think that these individuals were sicker or more likely to have different creatinine levels from others included.

Although there is little data for comparison, the relatively low prevalence of severe renal impairment seen here could reflect the fact that the local service is provided through a network of rural primary healthcare facilities reducing potential biases seen when collecting data from large centres, clinical trials or well organised urban cohorts which might recruit sicker or more complex patients.

Another possible explanation is that South African guidelines create a relatively high barrier to accessing antiretroviral treatment. Dedicated HIV services are often separate from hospital wards where many patients receive their first diagnosis and patients are required to undergo a period of education and training before treatment is started. Combined, these features might make it harder for sicker patients to start antiretrovirals. However, approximately one in seven HIV positive individuals in the world are living in South Africa and many, both in South Africa and beyond, are living in rural areas which means this data is likely to be representative of many populations starting antiretrovirals in Africa.

Other possible factors including circulating viral subtype, the prevalence of opportunistic infection and differences in human genetic structure, could also all contribute to differences in the prevalence of renal disease between African populations. It is interesting to note that in the recent study validating the MDRD estimation in South African blacks [10], the correction applied for African-Americans was not required, suggesting a differing genetic background with regard to renal disease between populations originating from Southern Africa and those from West Africa.

Nonetheless, information on the prevalence of conditions such as renal disease is valuable for the planning of antiretroviral services, particularly when there is a discussion about introducing tenofovir and where there are financial limitations for costs of both treatment and monitoring.

The costs of monitoring to prevent and detect toxicity from antiretrovirals depend not only on the prevalence of renal impairment but also whether costs can be reduced by inexpensive identification of a sub-group of patients who could be targeted for more intensive monitoring. It appears that in this setting, the use of routine simple urinalysis is not helpful in identifying an "at-risk" population.

Given the relatively small sample size for the assessment of urine analysis, these results need to be interpreted with caution. However, given what is known of the causes of renal diseases in the populations of Southern Africa, it is perhaps surprising that proteinuria in particular is not a more useful screening test.

Renal biopsy is not routinely available outside of a few specialist centres in South Africa and thus the causes of renal disease in this study cannot be stated with confidence. In a facility based series from Johannesburg, the aetiology of renal disease in HIV positive and HIV negative individuals differed significantly with HIV nephropathy (HIVAN), and HIV Immune Complex Kidney diseases (HIVICK) diagnosed in nearly half of all biopsies from HIV positive patients between 2003 and 2004[15]. These conditions are typically associated with proteinuria [16] as are the more common diseases of hypertension and diabetes, for which little other evidence was found in this population starting antiretrovirals despite a high prevalence locally[17]. There are different possible interpretations for this finding. One possibility is that intercurrent illness and acute tubular necrosis might be a more common aetiology in this population and most patients are symptomatic by the time they present for treatment. However, this conclusion cannot be sustained from the data here. Recently published data also suggests another possible explanation for the low levels of proteinuria detected. In a study from the US (with 94% African-American participants) urine dipsticks were found to be poorly sensitive to significant proteinuria detected by raised urine protein-creatinine ratios in HIV positive patients[18].

## Conclusion

Regardless of the underlying pathology these data do suggest that low cost tests such as urine analysis might not be sufficiently sensitive to be used as a single screening test for renal disease at baseline and there might not be an easy alternative to blood or urine biochemistry for screening patients initiating antiretrovirals in rural African populations. Further work will be needed to establish cost-effective means of identifying the small proportion of individuals with severe renal impairment, and larger studies to investigate this are required.

The extent to which this data is representative of the population at need of treatment in Southern Africa cannot be stated confidently, however, it is likely to represent a less biased population than many other sources. The data do suggest that renal impairment might not be as common as sometimes feared and that, in itself, it should not be seen as an impediment changes in the first line medication delivered through public health programmes in sub-Saharan Africa.

## Competing interests

The authors declare that they have no competing interests.

## Authors' contributions

CF, DK, GC were responsible for study design, data collection and analysis. TB, AA, RL, MD, MN provided input on analysis. All authors contributed to the manuscript and approved the final version.

## Pre-publication history

The pre-publication history for this paper can be accessed here:

http://www.biomedcentral.com/1471-2334/9/143/prepub
